# Successful Valve-in-Valve-in-Valve Transcatheter Aortic Valve Implantation for Severe Bioprosthetic Valve Restenosis in a High-Risk Patient

**DOI:** 10.7759/cureus.78805

**Published:** 2025-02-10

**Authors:** Benjamin A Gonzalez Burgos, Jose J Irizarry, Victor H Molina-Lopez, Juan Rivera-Torres, Miguel A Campos-Esteve, Antonio L Orraca-Gotay, Ismael Ortiz Cartagena

**Affiliations:** 1 Internal Medicine, Veterans Affairs Caribbean Healthcare System, San Juan, PRI; 2 Cardiology, Veterans Affairs Caribbean Healthcare System, San Juan, PRI; 3 Interventional Cardiology, Hospital Pavia, San Juan, PRI; 4 Cardiovascular Medicine, Pavia Santurce Hospital, San Juan, PRI; 5 Cardiovascular Disease, University of Florida Health, Jacksonville, USA

**Keywords:** bioprosthetic valve restenosis, hemodynamic improvement, high-risk surgical patients, minimally invasive valve replacement, new york heart association (nyha) class, sapien 3 valve, severe aortic stenosis, structural heart interventions, transcatheter aortic valve implantation (tavi), valve-in-valve-in-valve (viviv)

## Abstract

Transcatheter aortic valve implantation (TAVI) has significantly improved in treating aortic valve disease in recent years, particularly in patients at high surgical risk. This case report describes an 80-year-old woman who had severe aortic stenosis previously treated with surgical aortic valve replacement (SAVR) and six years later had a valve-in-valve (ViV) TAVI who developed severe symptomatic restenosis of the bioprosthetic aortic valve five years later of the last procedure. A third valve-in-valve-in-valve (ViViV) TAVI using a 26-mm Sapien 3 valve was performed due to the high surgical risk. The procedure resulted in significant hemodynamic improvement, reducing the transvalvular gradient from 80-90 mmHg to 15-20 mmHg and increasing the effective orifice area from 0.4 cm² to 1.5 cm². The patient’s symptoms improved to NYHA Class I. This case highlights the feasibility and safety of ViViV TAVI as a minimally invasive solution for recurrent bioprosthetic valve dysfunction in high-risk patients.

## Introduction

Bioprosthetic valves are increasingly favored for aortic valve disease due to their durability and safety profile. However, structural degeneration over time, leading to restenosis or regurgitation, poses challenges [1]. Transcatheter aortic valve implantation (TAVI) has evolved as an effective strategy for managing failed bioprosthetic valves, including in complex cases requiring repeat interventions such as valve-in-valve (ViV) or valve-in-valve-in-valve (ViViV) procedures [1]. The ViV technique extends this advancement, offering an effective solution for failed bioprosthetic valves. This procedure involves implanting a new valve inside a previously implanted bioprosthetic valve, offering a less invasive alternative with lower risks and complications compared to open-heart surgery, though it still carries its own procedural challenges. While ViV TAVI is widely used to treat failed bioprosthetic aortic valves, the ViViV procedure remains a rare and complex intervention, with only a few cases reported in the literature. It has been primarily utilized to correct severe paravalvular regurgitation (PVR) and to reduce transvalvular gradients in bioprosthetic aortic stenosis [2-4]. The ViViV procedure, though rare, exemplifies the potential for managing recurrent prosthetic valve dysfunction in a less invasive manner.

## Case presentation

An 80-year-old woman with a history of multiple aortic valve interventions presented with new onset worsening symptoms of heart failure (HF). Her medical history included myelodysplastic syndrome (MDS), hypertension (HTN), hyperlipidemia (HLD), chronic kidney disease (CKD) stage 3b, and prior surgical aortic valve replacement (SAVR) with a 21-mm Edwards Model Magna Ease 3300 TFX pericardial tissue valve in 2011 for severe aortic stenosis (AS). In 2020, she underwent TAVI with a 23-mm Medtronic Evolut R valve due to prosthetic valve stenosis. Over the past year, she experienced progressive dyspnea on exertion, orthopnea, and fatigue, consistent with New York Heart Association (NYHA) Class III heart failure symptoms. A transthoracic echocardiogram (TTE) revealed an LVEF of 40%, a mean gradient (MG) of 80-90 mmHg across the prosthetic valve, mild aortic regurgitation and an effective orifice area of 0.4 cm², indicating severe restenosis. TEE and CTA demonstrated significant calcification of the PrAV without evidence of thrombosis.

Given her age, comorbidities, and previous valve interventions, a multidisciplinary heart team decided to proceed with a ViV TAVI. The pre-procedural workup included coronary angiography, which ruled out significant coronary artery disease (CAD), and computed tomography (CT) TAVI to assess the annular size, valve positioning, and access route suitability. Blood tests showed stable hemoglobin at 11 g/dL (reference 12.6-17.8 g/dL), a creatinine level of 1.6 mg/dL (reference 0.7-1.5 mg/dL), and a lipid profile remarkable for LDL at 75.6 mg/dL (reference 0-100 mg/dL); all other lab results were within their reference ranges. The procedure was performed under TEE guidance and general anesthesia. The left radial artery was accessed using a 6-French (Fr) sheath to monitor arterial pressure and for coronary protection. The left femoral artery and vein were accessed with a 14-Fr Edwards sheath and a 5-Fr sheath for temporary pacing.

**Figure 1:**
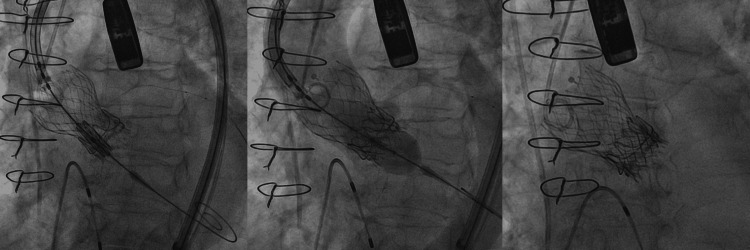
In the first frame the prosthetic valve delivery system is advanced into the previously placed bioprosthetic valves. The alignment and positioning are being carefully adjusted under fluoroscopic guidance to ensure optimal placement within the existing valve frames. In the second frame the valve is partially expanded, demonstrating precise alignment within the annular structure of the previously placed valves. The third frame shows the fully expanded third valve in position, with good apposition to the stent frames of the prior valves. The fluoroscopic image confirms the valve is securely deployed, with symmetrical stent expansion and no evident malposition.

**Figure 2:**
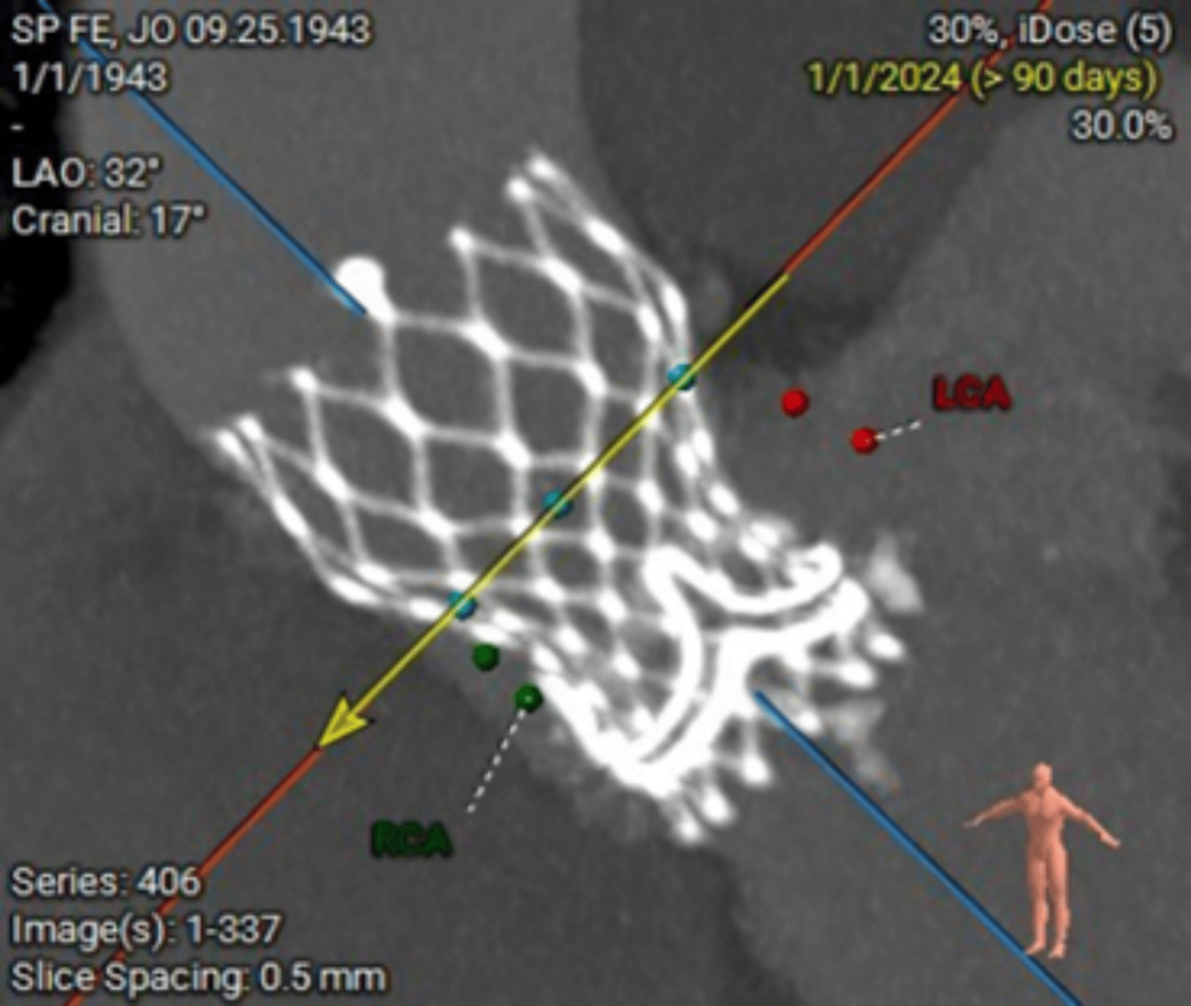
The sagittal view demonstrates a ViViV TAVI within the native calcified aortic annulus. The orientation of the prosthetic valve is visualized with a well-expanded stent frame. Markers indicate the coronary ostia’s proximity to the prosthesis, particularly the LCA. ViViV: valve-in-valve-in-valve; TAVI: transcatheter aortic valve implantation; LCA: left coronary artery

**Figure 3:**
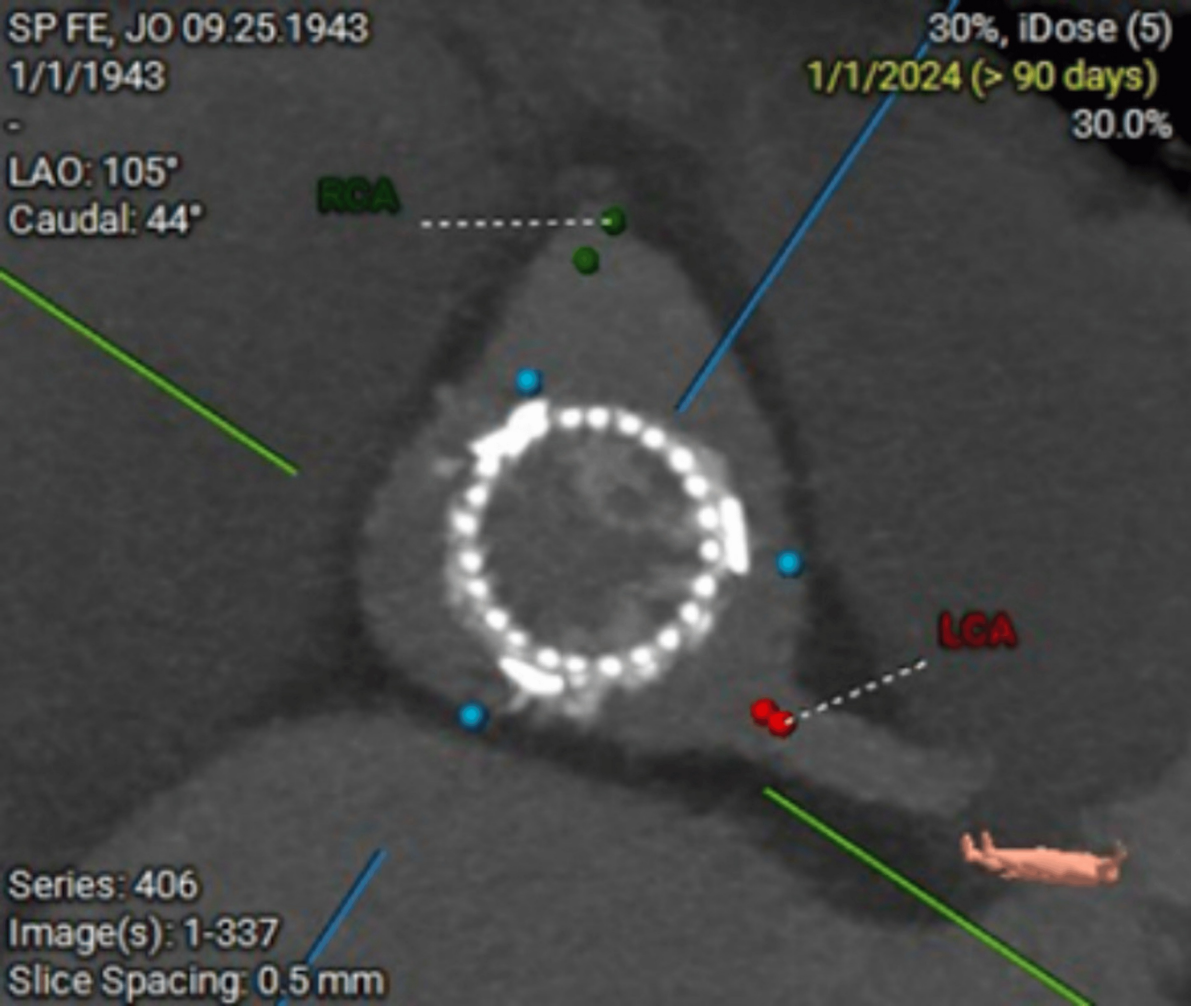
The cross-sectional view shows the tri-leaflet structure of the valve with excellent circularity, confirming proper deployment and seating. The distances to the RCA and LCA are measured, showing adequate clearance. RCA: right coronary artery; LCA: left coronary artery

**Figure 4:**
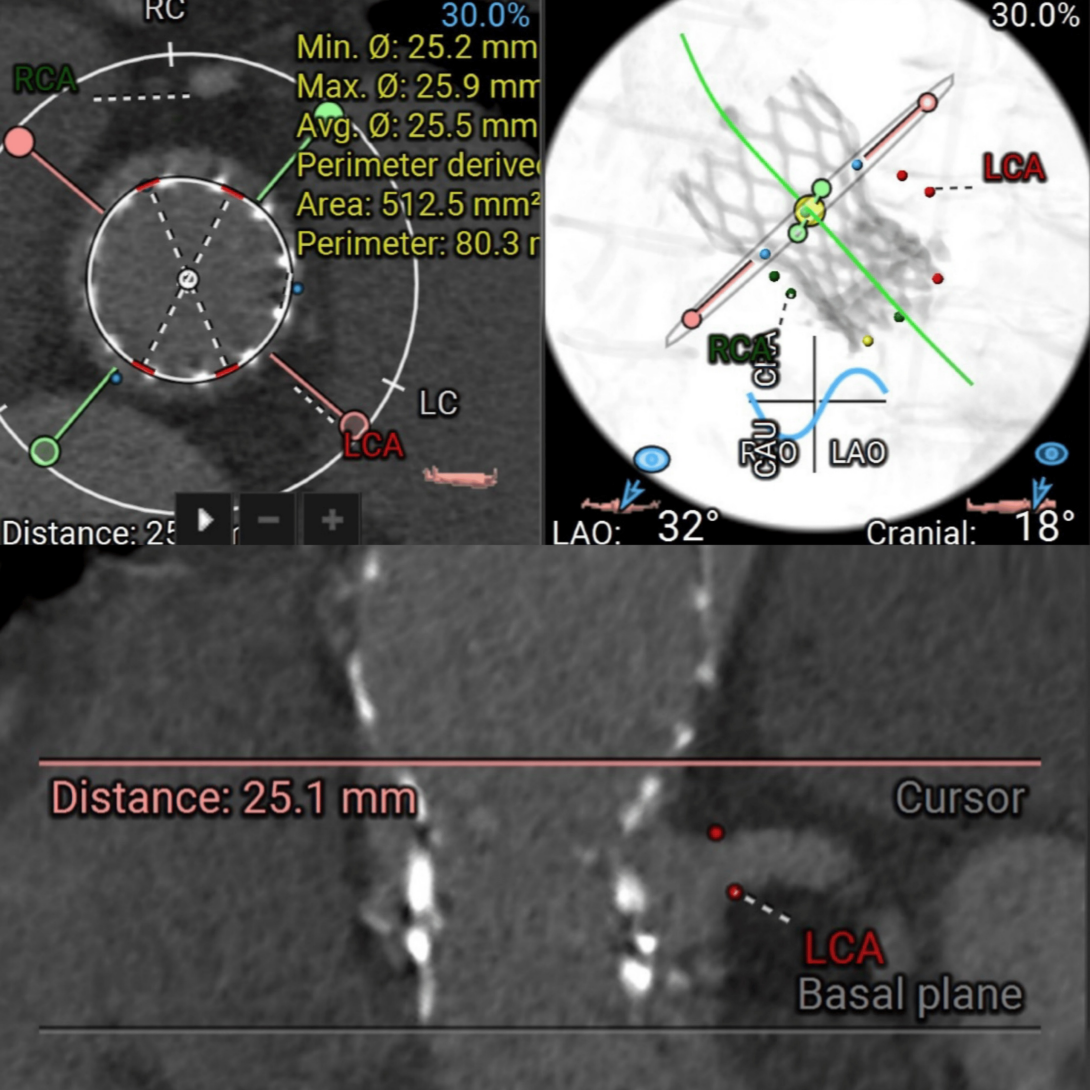
A detailed diagrammatic analysis illustrates the geometric assessment of the valve deployment. Measurements include minimum (25.2 mm) and maximum (25.9 mm) diameters, with a derived perimeter (80.3 mm) and area (512.5 mm²), confirming optimal prosthetic valve expansion. The proximity of the prosthesis to the LCA and RCA is also highlighted to evaluate coronary access post-procedure. RCA: right coronary artery; LCA: left coronary artery

Balloon aortic valvuloplasty (BAV) was performed with a 20-mm true balloon (non-compliant) to pre-dilate the stenotic prosthetic valve. A 20mmL +1mL (6.2% oversizing) Sapien Ultra Resilia valve was then advanced across the prosthetic valve and deployed under rapid pacing. Proper positioning was confirmed by fluoroscopy and TEE, and post-deployment angiography showed good valve expansion with no significant paravalvular leak. The valve was further post-dilated at +2 mL for 12.4% oversizing (Figure 1). The hemodynamic assessment demonstrated a significant and acceptable reduction in the transvalvular gradient from 80-90 mmHg to 15 mmHg and an improved effective orifice area from 0.4 cm² to 1.5 cm². No conduction disturbances were observed. There was no evidence of coronary obstruction post-implantation. Follow-up CT images reconstructed using 3mensio software demonstrated proper positioning of the aortic valve (Figures 2-4). Access sites were closed without complications.

Postoperatively, she remained hemodynamically stable without conduction abnormalities. Dual antiplatelet therapy with aspirin and clopidogrel was initiated to prevent thromboembolic complications. The patient was discharged home on postoperative day five with guideline-directed medical therapy (GDMT) for heart failure. Follow-up TTE at one month showed improved valve function with a mean gradient of 14 mmHg and an effective orifice area of 1.5 cm². Her symptoms improved to NYHA Class I.

## Discussion

Bioprosthetic valves, a common and effective solution for patients with aortic valve disease, face significant limitations due to structural deterioration over time. This deterioration manifests as regurgitation, restenosis, and valve degeneration [1]. As the use of bioprosthetic valves increases, there is a growing need for effective management strategies to address these complications, particularly in patients with failed bioprosthetic valves [5]. Repeated surgical aortic valve replacement (SAVR) has been the standard treatment for failed bioprosthetic valves. Still, it carries significant risks, including increased mortality, stroke, bleeding, length of stay, and recovery times, especially in older patients or those with multiple comorbidities [6-8]. As a result, the emergence of TAVI has shifted the management paradigm, offering a less invasive and safer alternative, particularly in high-risk patients.

TAVI has become the gold standard for treating severe native aortic stenosis, even in high-risk surgical patients. Its use is non-inferior to surgery in low- and intermediate-risk patients. One of the most significant advances in TAVI has been the development of the ViV technique, which allows for the implantation of a new transcatheter heart valve within a failing bioprosthetic valve. This technique has become a key therapeutic option for patients with high or prohibitive surgical risk [7-9]. Compared to repeat SAVR, ViV TAVR (transcatheter aortic valve replacement) has been associated with shorter recovery times, lower rates of bleeding and acute kidney injury, and generally better clinical outcomes [10-12].

The ViViV procedure is an exceedingly rare intervention that could become more relevant as patients who undergo ViV TAVI age, and their valves begin to fail. The idea of implanting a third valve into a failing ViV system was previously charted in a few cases, but none were related to severe valvular restenosis. This technique is currently only used in particular cases, typically involving patients with recurrent prosthetic valve dysfunction, such as paravalvular leaks (PVL), residual stenosis, or severe valve degeneration after a ViV procedure. Importantly, ViViV procedures often occur in patients who are already at high risk for complications due to advanced age, comorbidities, or previous surgical interventions [2-3].

**Figure 5:**
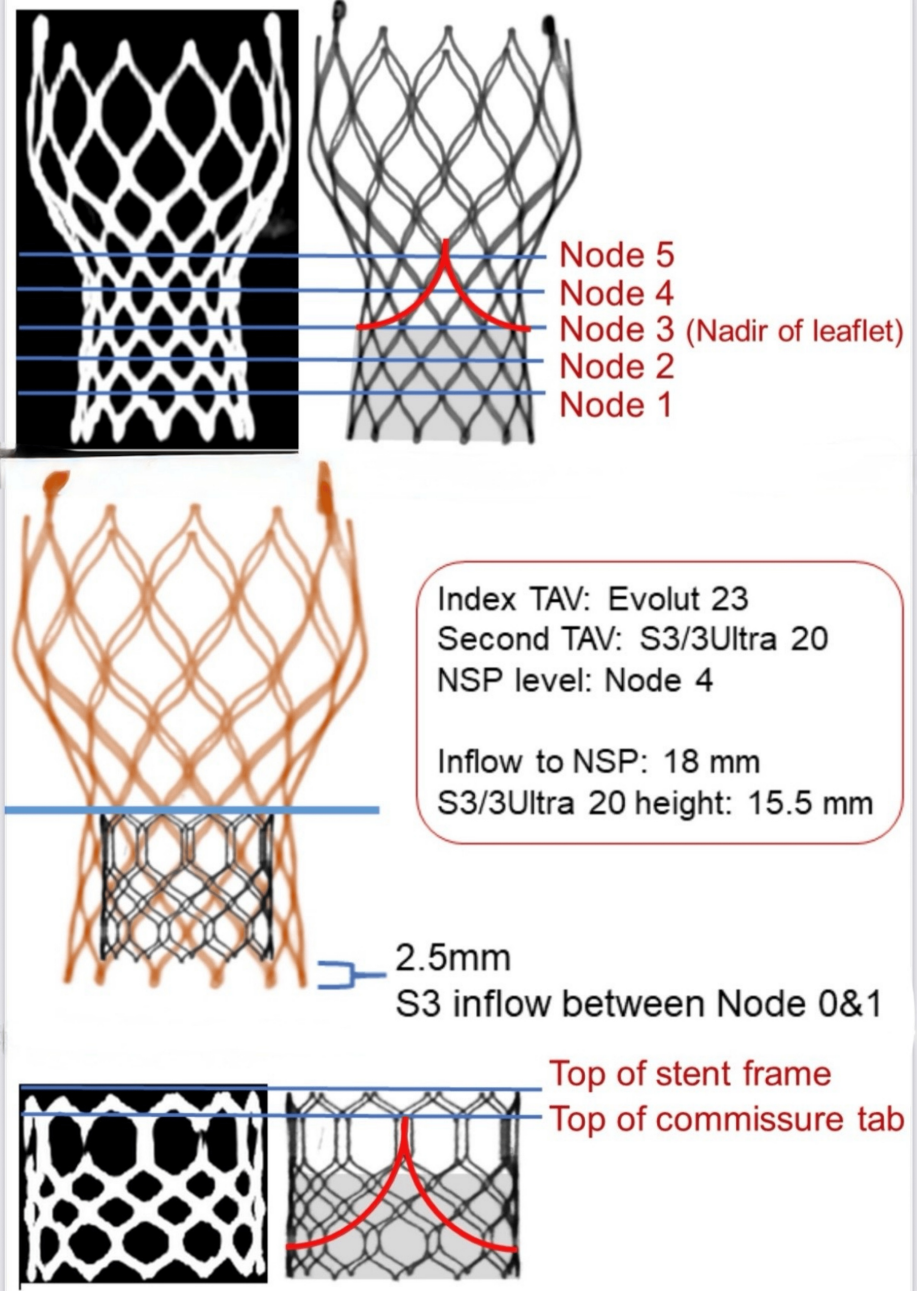
The image illustrates key structural and procedural aspects of a ViViV TAVI procedure, focusing on the alignment and interaction of the stent frames and valve components. The top panel depicts the Evolut stent frame with nodes labeled 1 to 5, identifying the nadir of the leaflet at Node 3. The middle panel overlays an Evolut 23-mm valve with a second Sapien S3/S3 Ultra 20-mm valve, highlighting the NSP at Node 4, with measured dimensions of 18 mm from inflow to NSP and a Sapien valve height of 15.5 mm. A clearance of 2.5 mm between the S3 valve inflow and the lower stent node is also noted. The bottom panel provides detailed views of the commissure tab and stent frame top, emphasizing leaflet positioning within the stent geometry. ViViV: valve-in-valve-in-valve; TAVI: transcatheter aortic valve implantation; LCA: left coronary artery; NSP: neo-skirt position This image is created by the authors of this study.

Several significant challenges arise when performing ViViV procedures, particularly when compared to initial ViV TAVI, including valve sizing and positioning, deliverability, coronary obstruction, and increased risk of valve thrombosis and degeneration [2-3]. In our case, we utilized a 20mmL +1mL (6.2% oversizing) Sapien Ultra Resilia Valve (Figure 5) to achieve a tight seal between the new valve and the existing one, thereby preventing PVL or valve displacement. Furthermore, the utilization of expandable valves is paramount for maintaining coronary artery access and enhancing the likelihood of future valve interventions should they become necessary [9, 11]. Long-term follow-up is essential to monitor for complications such as thrombosis, stent fractures, or residual regurgitation.

With increasing TAVI procedures performed worldwide, ViViV may become a more common approach to managing patients with failed ViV valves. As the number of patients requiring repeat interventions grows, ViViV procedures will likely become more standardized, with new techniques and technologies emerging to improve patient selection and procedural outcomes. It is essential to recognize that while ViViV offers a less invasive approach than repeat SAVR, it has its risks.

## Conclusions

In this case, we present a transcatheter ViViV procedure in a patient with a history of SAVR and a prior ViV TAVI. This innovative procedure highlights the evolving landscape of structural heart interventions and underscores the importance of personalized, multidisciplinary care. Although ViViV procedures are rare, they offer a valuable treatment option for patients with recurrent bioprosthetic valve dysfunction who are not candidates for surgical reoperation. As the population of TAVR-treated patients ages, ViViV may play an increasingly important role in managing patients with degenerating prosthetic valves. Continued research, advanced imaging techniques, and improved procedural strategies will be critical in optimizing the safety and efficacy of ViViV TAVI procedures.
